# The effect of step size on straight-line orientation

**DOI:** 10.1098/rsif.2019.0181

**Published:** 2019-08-07

**Authors:** Lana Khaldy, Orit Peleg, Claudia Tocco, L. Mahadevan, Marcus Byrne, Marie Dacke

**Affiliations:** 1 Department of Biology, Lund Vision Group, Lund University, Lund, Sweden; 2 Department of Computer Science, BioFrontiers Institute, University of Colorado Boulder, Boulder, CO, USA; 3 School of Animal, Plant and Environmental Sciences, University of the Witwatersrand, Johannesburg, South Africa; 4 Departments of Organismic and Evolutionary Biology and Physics, School of Engineering and Applied Sciences, Kavli Institute for NanoBio Science and Technology, Harvard University, Cambridge, MA, USA

**Keywords:** orientation, navigation, step size, dung beetle, compass, random walk

## Abstract

Moving along a straight path is a surprisingly difficult task. This is because, with each ensuing step, noise is generated in the motor and sensory systems, causing the animal to deviate from its intended route. When relying solely on internal sensory information to correct for this noise, the directional error generated with each stride accumulates, ultimately leading to a curved path. In contrast, external compass cues effectively allow the animal to correct for errors in its bearing. Here, we studied straight-line orientation in two different sized dung beetles. This allowed us to characterize and model the size of the directional error generated with each step, in the absence of external visual compass cues (*motor error*) as well as in the presence of these cues (*compass* and *motor errors*). In addition, we model how dung beetles balance the influence of internal and external orientation cues as they orient along straight paths under the open sky. We conclude that the directional error that unavoidably accumulates as the beetle travels is inversely proportional to the step size of the insect, and that both beetle species weigh the two sources of directional information in a similar fashion.

## 1. Background

To successfully locate mates, find food or escape predators or unfavourable environments, most animals need to travel along a given bearing in a relevant direction. For this, two main sources of directional information can be used; (i) information given by internal (proprioceptive) cues, for instance, by body rotations or leg movements [1–3], and (ii) information derived from external compass cues, such as the sun or the earth's magnetic field [4–8]. In practice, if an animal relies on internal mechanosensory information alone, it is not able to travel any greater distance from its current position (following a Brownian search where the distance travelled is proportional to the square root of the number of steps taken). This is clearly modelled by Cheung *et al*. [9]: an agent moving forward using only proprioceptive cues will, to some extent, with each successive step, deviate from the angular direction of the previous step taken. This is due to the accumulation of noise in the motor and sensory systems that will unavoidably result in the loss of ability to maintain the desired direction, subsequently making straight-line orientation impossible. This has been demonstrated behaviourally in animals as diverse as humans, spiders and beetles [10–12].

In most navigating and migrating animals, directional guidance is acquired from multiple sources of information [13–16], originating from the movement of the animal itself (*internal cues*) [1–3] and/or from its surroundings (*external cues*) [17–19]. Experimental studies from species as diverse as ants [16], butterflies [20], elk [21] and grey seals [22] have considered how animals may balance these two sources of directional information to navigate within their local environment. In ants, different sources of directional information are weighted relative to their respective certainty, where the cue conveying the highest certainty is afforded the highest weight in the directional output from the compass [16,23,24]. For instance, if the visual scenery fails to provide the information needed, the ant will rely more strongly on the directional information provided by its path integrator to complete its navigational task [16].

In contrast to most migrating and homing animals, a ball-rolling dung beetle simply needs to move along one single bearing for the duration of its current travels. At the dung pat—where the journey of a ball-rolling beetle begins—competition for dung can be fierce [25]. In order to obtain a sufficient amount of food, beetles gather and shape dung into balls and roll them away. To ensure the most efficient escape from the competitors at the pat, the beetles exit in all directions along paths as straight as the terrain allows [6,26–29]. In this way, with every step taken, they maximize the distance between themselves and their competitors. For diurnal beetles, the most prominent compass cue used to steer along this set bearing is the sun [6,27,28,30]. Together with the celestial polarization pattern, the skylight intensity gradient and the colour gradient across the sky, this forms a highly robust straight-line orientation system [30–32]. If these cues are eliminated from the ball-rolling dung beetle's field of view, the animal soon loses its ability to maintain a straight bearing [12].

Understanding how animals balance internal and external directional cues to maintain a straight bearing is still an open question in movement ecology. It is challenging to model behaviour in the ecological context of homing insects [16,20], elks [21] and seals [22] that forage within a familiar territory. This is because most theoretical models assume a straight-line optimal trajectory [33], while the actual trajectories travelled by these animals may be more tortuous. In contrast, ball-rolling dung beetles strive to move along straight paths from the start to the end of their journeys [6,12,27–30]. Here, we characterize the size of the directional error generated with each step in the presence or absence of external compass cues in two closely related species of dung beetle, that differ greatly in size. This allows us to (i) estimate the influence of step size on the precision of straight-line orientation [34,35] and (ii) model the weight given to external sky compass cues over internal proprioceptive cues for straight-line orientation in dung beetles.

## 2. Methods

### 2.1. General

Two closely related species of diurnal dung beetles, *Scarabaeus* (*Scarabaeus*) *ambiguus*, and *Scarabaeus* (*Kheper*) *lamarcki* were collected within the elephant park Adventures with Elephants (27.95°E, 24.78°S) and Stonehenge game farm (24.32°E, 26.39°S), respectively, in South Africa with the aid of dung-baited pitfall traps. Experiments were performed at Stonehenge game farm and Thornwood lodge (28.02°E, 24.77°S), during February 2017. All experiments (apart from the studies of orientation performance in the absence of visual cues which were conducted in complete darkness in a light-tight indoor laboratory) were performed outdoors under clear skies at solar elevations between 45 and 60°. Each beetle was marked individually with a number on its thorax using a white marker (Tipp-Ex^®^). An overhead Sony Handycam HDR-CX730E (fitted with a 0.42× wide angle lens to extend the field of view when required) was used to record dung beetle rolling trajectories and exit bearings.

### 2.2. Step size determination

**Figure 1.**
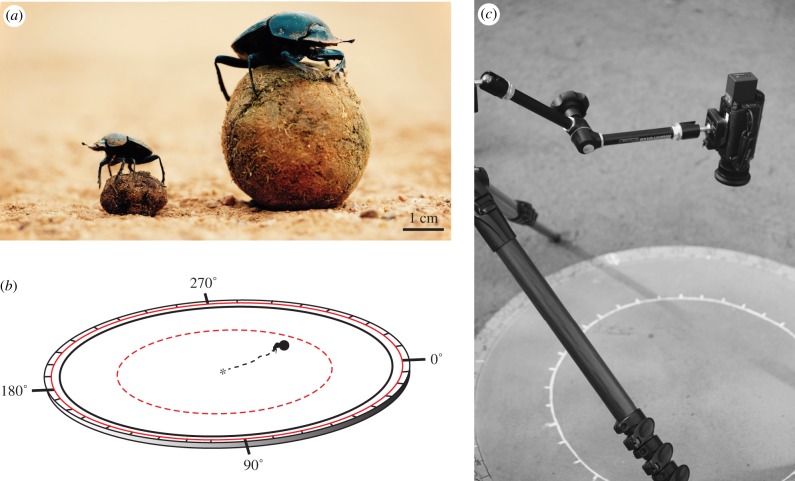
Description of the experimental arenas and beetles. Individuals of *Scarabaeus ambiguus* (left) and *S. lamarcki* (right) are depicted side-by-side for size comparison. *Photo: Christopher Collingridge* (*a*). For all treatments, a beetle was placed alongside a ball in the centre of a circular, sand-coated arena (*b*) and filmed with an overhead camera (*c*). The beetle was allowed to roll its ball to the perimeter of the arena, where the exit angle was noted (0° = magnetic North). Depending on the experimental treatment, the beetle either rolled only once, or was repeatedly placed back in the arena centre beside its ball until it had exited the arena 20 times. The ball rolled was either a natural dung ball or a standard putty ball (shown in *a*). For the experiment where the sun was mirrored, a 75 cm radius arena was used (not depicted in the figure). For all other experiments conducted, three differently sized arenas were used depending on the species of beetle (*b*): 50 cm (*S. ambiguus* and *S. lamarcki*, black solid line), 33 cm (*S. ambiguus*, red dotted inner circle), 52 cm (*S. lamarcki*, red solid outer circle) radius. (Online version in colour.)

Individual beetles were allowed to roll their dung ball across a flat, sand-coated (Dried Ochre, granular paint, Fired Earth™), 50 cm radius, outdoor arena (figure 1*a*). Step size was determined as the distance from the point at which the front foreleg (left or right) was stable on the arena surface, to the point at which the same limb was again stable on the surface. Image processing software, ImageJ1© (National Institutes of Health, Bethesda, MD, USA), was used to extract the *x* and *y* coordinates of the start and endpoint of each step from the overhead videos. From this, the length of the step was calculated and converted to true length by using a millimetre scale present in the frame for reference. The step length for each species was determined by calculating the average length of 10 strides per beetle for each species (*N* = 10).

### 2.3. The role of the sun in the celestial compass system of the two beetle species

Beetles were placed individually alongside their dung ball in the centre of a flat, circular, sand-coated, wooden, outdoor arena, measuring 75 cm in radius, and allowed to roll the ball to the edge, where the exit bearing was noted. The beetle was then removed from its ball and placed back in the centre of the arena alongside its ball. At the same time, the sun's apparent position was changed by 180°, using a mirror (30 × 30 cm), while simultaneously concealing the real sun from the beetle's field of view with a wooden board (100 × 75 cm). Again, the beetle was allowed to roll to the edge of the arena and its second exit bearing was noted. A third trial, with an un-manipulated sun position as in the first trial, was performed as a control to verify that the beetle strived to adhere to approximately the same bearing throughout the experiment. This held true for all beetles tested. In total, 45 individuals per species rolled from the centre to the edge of the arena three times.

**Figure 2.**
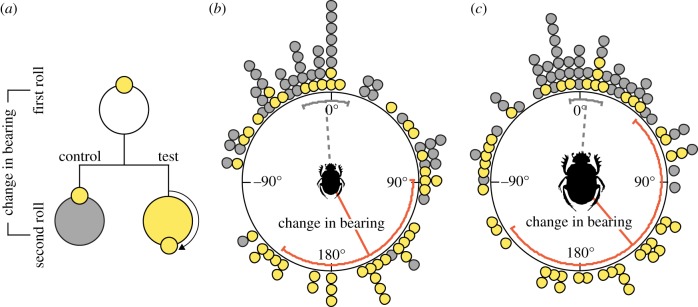
The role of the sun in the celestial compass system of two beetle species. The response to a mirrored sun while rolling outdoors, under a clear sky, was tested in two closely related, but differently sized beetle species. A schematic diagram of the experiment is presented in (*a*). Forty-five individuals of *Scarabaeus ambiguus* (*b*) and *S. lamarcki* (*c*), respectively, were individually placed under the natural sky, alongside a dung ball in the centre of a 75 cm radius, circular sand-covered arena. The beetles were allowed to roll to the perimeter of the arena where their exit angles were noted. From here, the beetles were placed back in the centre to exit a second time, either under (i) the same natural sky as during the previous roll (*control*, grey circles), or (ii) a manipulated sky where the apparent position of the sun is changed by 180° by the use of a mirror (*test*, yellow circles). The difference between two exit angles was calculated and used to define the mean change in bearing (*control*, dotted grey lines; *test*, solid red lines). Error bars represent one circular standard deviation. When allowed to roll twice under the sun, individuals of both species showed no significant change in bearing between consecutive rolls (dotted grey line). Under the mirrored sun, both species responded to this treatment by a change in exit bearing approaching 180° (solid red lines). (Online version in colour.)

Angular change was calculated as the difference in exit bearing between two exits from the arena (figure 2). Directional statistics were obtained from Oriana 3.0 (Kovach Computing Services, Anglesey, UK).

### 2.4. The effect of step size on orientation performance

To eliminate any influence of size or shape of beetle-made dung balls on the orientation performance of the different beetles, ‘standard balls’ were made from dung infused Play-Doh^®^ (Hasbro, Pawtucket, RI, USA). These balls had a set size of 1.7 cm diameter for *Scarabaeus ambiguus* and 3.5 cm diameter for *S. lamarcki* (figure 1*a*). These dimensions were determined from the average diameter of beetle crafted balls for each species (*S. ambiguus*: 1.74 ± 0.13 cm (mean ± s.e.m.), *S. lamarcki*: 3.54 ± 0.60 cm) (*N* = 10).

#### 2.4.1. The effect of step size on orientation performance in the absence of external visual cues

**Figure 3.**
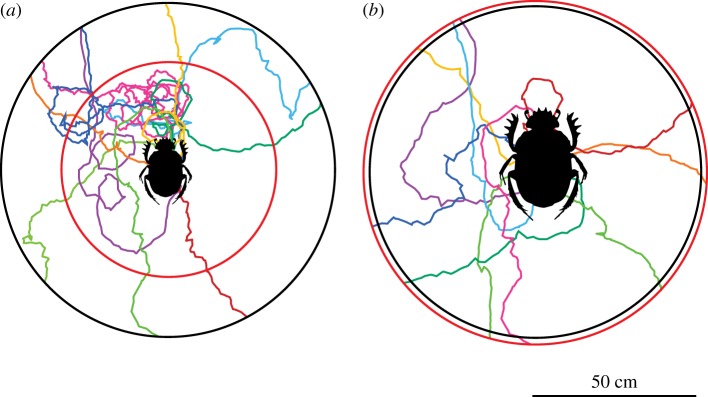
Rolling trajectories in the absence of external visual cues. The two closely related, but differently sized beetles, were allowed to roll a dung ball from the centre of a flat, sand-coated arena, in complete darkness. The full trajectories of 10 randomly chosen beetles of each species are shown. On the 50 cm radius arena (black perimeter) *S. ambiguus* (*a*), obtained a significantly lower *straightness index* (higher tortuosity) compared to the larger *S. lamarcki* (*p* = 0.02, *N* = 15) (*b*). When analysed over a radial distance corresponding to 20 steps for each species respectively (32 cm for *S. ambiguus* and 52 cm for *S. lamarcki*) (*a*, inner red perimeter; *b*, outer red perimeter) no significant difference in straightness was recorded (*p* = 0.08, *N* = 15). (Online version in colour.)

In these experiments, beetles were individually placed beside a ‘standard ball’ of the size associated with their species (figure 1*b*), at the centre of a flat, circular, sand-coated, wooden, arena, measuring 50 cm in radius in the complete darkness. From here, the beetle rolled the ball to the perimeter of the arena. This marked the end of the trial (figure 3). To record the beetles' trajectories in the dark, the overhead camera was fitted with an infrared lamp, and individuals were marked with high-gain reflective paint (Soppec©: Technima Nordic AB, Mölndal, Sweden) on their thorax. In total, 10 individuals per species were tested.

The beetle's position in each video frame was determined using custom-made tracking software in Matlab R2016a (Mathworks Inc., Natwick, MA, USA, courtesy of Dr Jochen Smolka). Camera calibration software in Matlab was used to correct for optical distortion and true distances were obtained from a calibration pattern (3 × 3 cm black and white squares) placed on the surface of the arena. Path length of each roll was calculated by summing the two-dimensional distance travelled between successive frames. To determine how straight the beetle's trajectories were, a *straightness index* (*S*) was calculated as *D/W* [36], where *D* is the distance from the starting point to the perimeter of the arena, and *W* is the length of the path taken.

**Figure 4.**
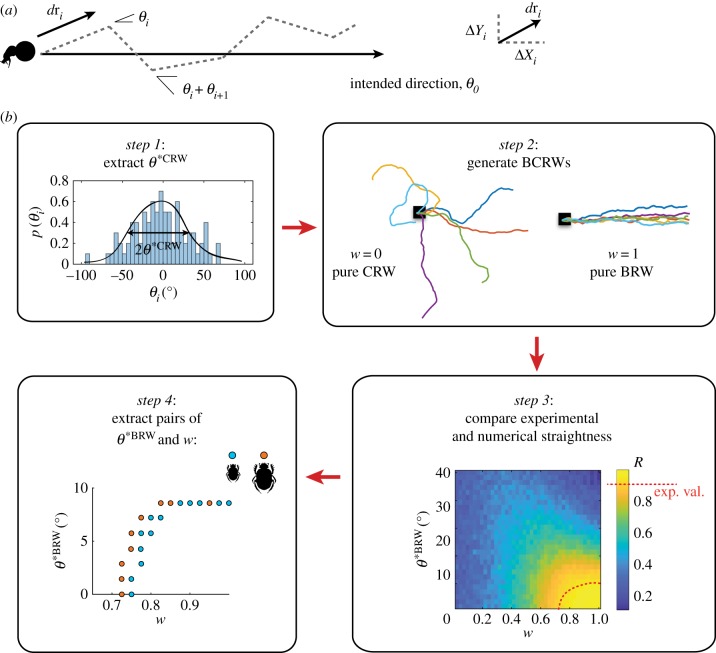
Estimation of motor errors, compass errors, and their balance. A model of a beetle performing a random walk where *θ_i_* is the direction of movement of the previous step and Δ*X_i_*, Δ*Y_i_* are the distance travelled in step *i* along the *x* and *y* directions, respectively (defined in equations (2.1) and (2.2)) (*a*). A flow diagram describing the process of estimating pairs of *w* and θ^*BRW^ for two beetle species that differ in size (*b*). *Step 1*: θ^*CRW^ is estimated from the width of the angular errors of a beetle orienting in the absence of visual cues. *Step 2*: Two sets of BCRW trajectories are illustrated; one at the limit of pure CRW (*w* = 0) and one at the limit of pure BRW (*w* = 1). To generate these trajectory examples, we chose arbitrarily θ^*BRW^ = θ^*CRW^ = 5° (these values were arbitrarily chosen for the purpose of illustrating the model). Each trajectory is shown in a different colour. *Step 3*: Mean vector length (*R*) for each species is generated from the simulation and compared to the experimentally measured values (shown as red dotted line on the colour bar and on the heat-map). *Step 4*: The extracted *w* and θ^*BRW^ for each species is shown. (Online version in colour.)

In order to determine the angular error generated by each step in the absence of external visual cues, 15 individuals of each species were filmed at close range, with an overhead camera fitted with an infrared lamp. Using a custom-made tracking software in Matlab 2017b (Mathworks Inc. Natwick, MA, USA), the angular error generated by each step of the beetle, was determined. For this, we defined a step as the instance of foreleg-surface contact. The position of two consecutive surface contacts by the same foreleg was tracked and a vector between these two consecutive points was drawn to determine the bearing direction of one step. From this, angular error per step was calculated as the absolute difference in bearing direction between two consecutive vectors (figure 4*a*).

#### 2.4.2. The effect of step size on orientation performance in the presence of external visual cues

To determine the orientation performance of the two beetle species under an open sky, each beetle, together with a species specific ‘standard ball’, was repeatedly placed in the centre of a flat, circular, sand-coated, wooden, outdoor arena, until each beetle had rolled its ball to the edge of the arena 20 times. Two different sized arenas were used (figure 1*c*); (i) one with the effective radius set to a distance equivalent to the length of 20 steps for the species tested (*S. ambiguus*: 32.38 cm, *S. lamarcki*: 51.79 cm) and (ii) one with a radius of 50 cm. The exit bearings of 20 rolls performed by each beetle were again noted and all trajectories were recorded from above. In total, 20 individuals per species were tested.

**Figure 5.**
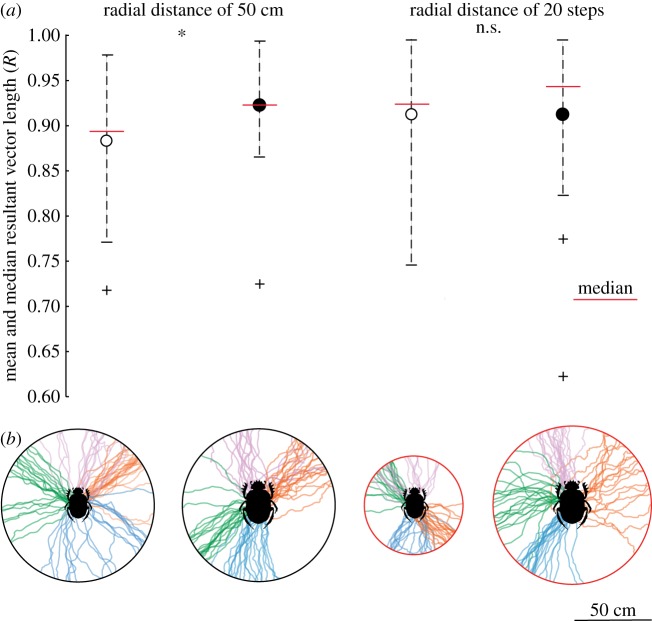
Measuring orientation performance in the presence of external visual cues. As a measure of orientation performance (*a*), the mean vector length for each beetle was calculated from 20 tracks over a radius equivalent of 50 cm, as well as of a radius equivalent of 20 step lengths of the corresponding species (32 cm for *Scarabaeus ambiguus and* 52 cm for *S. lamarcki*) (white circle, mean value for *S. ambiguus*; black circle, mean value for *S. lamarcki*; red solid line, median value for *S. ambiguus* and *S. lamarcki*). An *R*-value of 1 indicates that the beetles maintained the same direction over 20 rolls. When rolling over a radius of 50 cm, the smaller species, *S. ambiguus* (*N* = 20), showed a significantly shorter resultant vector length compared to the larger species (*N* = 20) (*R_S. ambiguus_*: 0.88 ± 0.02; *R_S. lamarcki_*: 0.92 ± 0.01, *p* = 0.028, *N* = 20). However, no significant difference was seen when both species rolled over a distance equivalent to 20 steps (*R_S. ambiguus_*: 0.91 ± 0.015; *R_S. lamarcki_*: 0.91 ± 0.02, *p* = 0.42, *N* = 20). Paths travelled by four individuals for each species and radial distance (*b*) are shown (from left: *S. ambiguus* (50 cm); *S. lamarcki* (50 cm); *S. ambiguus* (32 cm); *S. lamarcki* (52 cm)). Each colour represents 20 trajectories of one individual. * = *p* < 0.05, n.s. = *p* > 0.05. There was no difference in the straightness of the 20th exit path compared to the 1st exit path performed by the same individual in any of the conditions (*p*_50 cm(*S. ambiguus*)_ = 0.16, *p*_50 cm(*S. lamarcki*)_ = 0.16; *p*_step length(*S. ambiguus*)_ = 0.30, *p*_step length(*S. lamarcki*)_ = 0.16, *N* = 20). (Online version in colour.)

Orientation performance of each individual was determined by the mean resultant vector length (*R*) calculated in Oriana 3.0 (Kovach Computing Services, Anglesey, UK) from the 20 exit bearings for one individual (figure 5). This value is used to describe the concentration of unimodal circular distribution, and ranges from a value of 0 (random distribution of angles) to a value of 1 (no dispersion in distribution of angles). The better oriented the beetle, the closer the exit bearings cluster around one direction, and the closer the mean resultant vector is to unity. In total, the orientation performance of 20 individuals per species for the treatments described above were recorded.

### 2.5. Estimation of motor errors, compass errors, and their weight

The integration of proprioceptive cues in the orientation system of an agent generates noise (termed *motor error*) affecting the motoric output of the agent. Similarly, the detection and integration of external compass cues by the orientation system generates noise (termed *compass error*). Both of these sources of noise can be expected to affect the motoric output of an agent exercising straight-line orientation.

The *biased correlated random walk model* [35] was used to estimate the compass error of external cues and determine how much weight is given to external visual cues over internal proprioceptive cues for straight-line orientation in the beetles. In both *biased* and *correlated random walks*, the agent's goal is to walk in a straight line in an arbitrary direction *θ*_0_ (figure 4*a*). In a biased random walk (BRW) the instantaneous angular error, *θ_i_*, arises from noise in external visual cue acquisition (*compass error*), however, in a correlated random walk (CRW) it arises from accumulated noise in motoric execution (*motor error*). The *biased correlated random walk model* combines the two errors in the following manner:

2.1 $$\Delta X_{i+1}=l[w\cos(\theta_{0}+\theta_{i}^{\mathrm{BRW}})+(1-w)\cos(\theta_{i}+\theta_{i}^{\mathrm{CRW}})]$$

and

2.2 $$\Delta Y_{i+1}=l[w\sin(\theta_{0}+\theta_{i}^{\mathrm{BRW}})+(1-w)\sin(\theta_{i}+\theta_{i}^{\mathrm{CRW}})]$$

where *l* is the step length of the current step, *θ_i_* is the direction of movement of the previous step, $\theta_{i}^{\mathrm{CRW}}\;$is an motor error term, $\theta_{i}^{\mathrm{BRW}}$ is an compass error term, and *w* ∈ [0, 1] is the weighting given to external cues. We assume that $\theta_{i}^{\mathrm{CRW}}$ and $\theta_{i}^{\mathrm{BRW}}$ are random angles drawn from a von Mises distribution with a zero-mean and standard deviation θ^*CRW^ and θ^*BRW^, respectively. Table 1 summarizes the model parameters and their experimental equivalents. θ^*CRW^ was estimated for each species from the angular errors experimentally measured in the absence of visual cues and used as input parameters to the model (figure 4*b*, step 1). BCRW trajectories with the estimated value of θ^*CRW^ were generated numerically (*N* = 500) with a range of values for *w* and θ^*BRW^ (figure 4*b*, step 2). The resulting mean vector length, *R*, was compared against the experimentally measured *R* (figure 4*b*, step 3) to estimate pairs of *w* and θ^*BRW^ for the two different sized beetle species (figure 4*b*, step 4). Due to rotational symmetry, *R* is independent of the direction *θ*_0_, hence we arbitrarily set its value to zero.

**Table 1.** Biased correlated random walk model parameters.

parameter	meaning	experimental value	theoretical value (model inputs/outputs)
*l*	step size	extracted from step analysis of beetles in the presence of external visual cues	set by the experimental values *S. ambiguus*: *l* = 1.6 cm *S. lamarcki*: *l* = 2.6 cm
*θ*_0_	intended direction	extracted as mean exit bearing	0 (towards the right)
*w*	balance between CRW and BRW	unknown	extracted to fit properties of experimental trajectories (*R*): *S. ambiguus*: $\begin{array}{rl} \theta^{*\mathrm{BRW}}=\; & 5.95^{\circ }\pm 2.97^{\circ }\;\;\\ w=\; & 0.84\;\;\pm \;\;0.09 \end{array}$ *S. lamarcki*: $\begin{array}{rl} \theta^{*\mathrm{BRW}}=\; & 6.34^{\circ }\pm 3.01^{\circ }\\ w=\; & 0.83\;\;\pm \;\;0.08 \end{array}$
θ^*BRW^	standard deviation of compass error	unknown	
θ^*CRW^	standard deviation of motor error	extracted from step analysis of beetles in the absence of external orientation cues	set by the experimental value *S. ambiguus*: θ^*CRW^ = 33.11° ± 5.12° [*N* = 5] *S. lamarcki*: θ^*CRW^ = 29.41° ± 9.92° [*N* = 10]

### 2.6. The role of step size in the natural habitat

**Figure 6.**
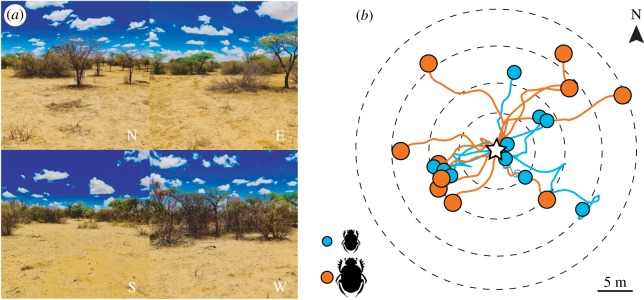
Orientation performance in a natural environment. The smaller *Scarabaeus ambiguus* and the larger *S. lamarcki* were allowed to form a dung ball and roll it away from a dung pat (marked with a star) in their natural environment (N, north; E, east; S, south; W, west) (*a*). Their trajectories (blue lines, *S. ambiguus*; orange lines, *S. lamarcki*), were recorded until they started to bury their balls (blue circles, *S. ambiguus*; orange circles, *S. lamarcki* (*b*)). This marked the end of the trial and the radial distance from the dung pat to the site of burial was measured. The dotted concentric circles indicate radial distances from the dung pat in 5 m increments. In total, trajectories of 10 individuals per species were recorded. The smaller species, *S. ambiguus*, rolled a significantly shorter radial distance from the pat before burying its ball when compared to the larger species (*S. ambiguus*: 7.56 m ± 1.05 m, *S. lamarcki*: 12.45 m ± 1.28 m, *N* = 10) (*p* = 0.001, *N* = 10). (Online version in colour.)

Ten individuals of each species were allowed to sculpt and roll a dung ball from a pat of 1 l cow dung, placed in the savannah on a sunlit day (figure 6). Trials alternated between the two species. The paths of the beetles were recorded by a hand-held video camera (GoPro^®^ HERO5 Black), held approximately 1 m above the beetle as it rolled, until the beetle started to bury its ball. Bearing direction and distance from the centre of the dung pat to the site of burial were then measured. Positional information for the individual segments of the path trajectories from beetles rolling across this natural terrain was obtained using a tailor-made analysis tool [37] (courtesy of Dr Benjamin Risse, University of Münster). To extract shape and total distance travelled for each trajectory, a custom-made Matlab script was used.

## 3. Results

### 3.1. Body size and step size determination

The two closely related, ball-rolling species of South African dung beetles, *Scarabaeus ambiguus* and *S. lamarcki* differ significantly in size with a pronotum width of 1.09 ± 0.01 cm and 2.07 ± 0.03 cm and a body length (tip of abdomen to tip of pronotum) of 1.52 ± 0.01 cm and 2.86 ± 0.04 cm (mean ± s.e.m. *N* = 10) respectively (Wilcoxon rank-sum test; *p*_Pronotum_ < 0.001, *p*_Body length_ < 0.001, *Z*_Pronotum_ = −3.75, *Z*_Body length_ = −3.76). Not surprisingly, the average step size for the smaller *S. ambiguus* (1.69 ± 0.09 cm, *N* = 20), is significantly shorter than that of the larger *S. lamarcki* (2.89 ± 0.08 cm, *N* = 20) (Wilcoxon rank-sum test; *p* < 0.001).

### 3.2. The role of the sun in the celestial compass system of the two beetle species

To investigate the role of the sun in the compass system of the two species, the response of an orienting beetle in terms of change in roll bearing, was tested under an un-manipulated sky as well as under a sky with the position of the sun changed by 180° by the use of a mirror (and the real sun simultaneously shielded from view of the beetle). When the position of the sun was artificially changed by 180° (*test*) on the second roll, both species changed their headings in response to this manipulation, with the same order of magnitude (*μ_S. ambiguus_* = 152.37° ± 105.29°, *μ_S. lamarcki_* = 139.39° ± 117.45°, Mardia–Watson–Wheeler test; *p* = 0.92, *N* = 45, W = 0.16) (figure 2). No significant change in direction (*μ*) was seen in individuals of either of the two species of beetles between two rolls made under an unobscured sky (*control*) (*μ_S. ambiguus_* = −3.61° ± 53.56° (mean ± s.d.), *μ_S. lamarcki_* = 4.43° ± 38.61°, *V*-test (with the expected mean of 0°); *p_S. ambiguus_* < 0.001, *p_S. lamarcki_* < 0.001, *N* = 45, *V_S. ambiguus_* = 0.65, *V_S. lamarcki_* = 0.79). This demonstrates that both species orient using a sun compass.

### 3.3. Orientation performance in the absence and presence of external visual cues

#### 3.3.1. In the absence of visual cues

When rolling over a radial distance equivalent to the length of 20 steps of the respective species (*S. ambiguus*: 32.38 cm, *S. lamarcki*: 51.79 cm, see Methods, figure 1) there was no significant difference between the straightness of the trajectories travelled by the two species (*S_S. ambiguus_* = 0.48 ± 0.12; *S_S. lamarcki_* = 0.62 ± 0.16, Wilcoxon rank-sum test; *p* = 0.08, *Z* = −0.79) (figure 3). When instead analysing the straightness of tracks across a radial distance of 50 cm, the smaller species, *Scarabaeus ambiguus* had a lower *straightness index* (*S*) compared to the larger *S. lamarcki* (*S_S. ambiguus_* = 0.45 ± 0.12; *S_S. lamarcki_* = 0.65 ± 0.17 (mean ± s.e.m., *N* = 15), Wilcoxon rank-sum test; *p* = 0.02, *Z* = 2.32) (figure 3).

This indicates that the size of the angular error generated by each step, θ^*CRW^, does not differ between the two species of beetles. This was further confirmed from the direct comparison between species (*S. ambiguus*: 33.11° ± 5.12°, *N* = 5; *S. lamarcki*: 29.41° ± 9.92°, *N* = 10, Wilcoxon rank-sum test; *p* = 0.86, *U* = 42).

#### 3.3.2. In the presence of visual cues

When rolling 20 times across an arena with a radius proportional to 20 step lengths of the two species (*S. ambiguus*: 32.38 cm, *S. lamarcki*: 51.79 cm), we found no significant difference in mean resultant vector length (i.e. spread of exit bearings) between the different sized beetles (*R_S. ambiguus_*: 0.91 ± 0.015; *R_S. lamarcki_*: 0.91 ± 0.02, Wilcoxon rank-sum test; *p* = 0.42, *N* = 20, *Z* = −0.83) (figure 5). When instead testing the orientation performance over a radius of 50 cm, *Scarabaeus ambiguus* showed a significantly shorter mean resultant vector length compared to that of *S. lamarcki* (*R_S. ambiguus_*: 0.88 ± 0.02; *R_S. lamarcki_*: 0.92 ± 0.01, Wilcoxon rank-sum test; *p* = 0.028, *N* = 20, *Z* = −2.20) (figure 5). This indicates that *S. ambiguus*, the smaller of the two species, is less able to maintain its bearing over a given distance compared to its larger relative when rolling under a natural sky.

We found no difference in the straightness of the 20th exit path compared to the 1st exit path performed by the same individual (Wilcoxon rank-sum test; *p*_50 cm(*S. ambiguus*)_ = 0.16, *Z* = 1.42; *p*_50 cm(*S. lamarcki*)_ = 0.16, *Z* = 1.42; *p*_step length(*S. ambiguus*)_ = 0.30, *Z* = 1.04; *p*_step length(*S. lamarcki*)_ = 0.16, *Z* = 1.42, *N* = 20). This suggests that the error generated with each step does not change in size with distance rolled but remains the same regardless of the number of steps taken.

### 3.4. Weighting of compass and motor errors

The angular error generated by each step in the absence of external visual compass cues was introduced as an estimation of motor error (θ^*CRW^) in the *biased correlated random walk model* [36] (figure 4*b*, step 1). This allowed us to compare the resulting mean vector length, *R*, of the modelled data against the experimentally obtained *R* values (figure 4*b*, step 3) to estimate pairs of *w* (the balance between CRW and BRW) and θ^*BRW^ (standard deviation of compass error) for the two species (figure 4*b*, step 4). From this, no significant difference was found in the balance between *CRW* and *BRW*, between the two species (*w_S. ambiguus_*: 0.84 ± 0.09, *w_S. lamarcki_*: 0.83 ± 0.08; Wilcoxon rank-sum test; *p* = 0.54, *N* = 13, *U* = 183). This also held true for the mean compass error θ^*BRW^ ($\theta_{S.\;ambiguus}^{*\mathrm{BRW}}:\;5.95^{\circ }\;\pm \;2.97^{\circ },$ $\theta_{S.\;lamarcki}^{*\mathrm{BRW}}:\;6.34^{\circ }\;\pm \;3.01^{\circ },$ Wilcoxon rank-sum test; *p* = 0.65, *N* = 13, *U* = 205.5). This suggests that the relative balance between internal and external compass cues for straight-line orientation in beetles is not affected by differences in stride length. This finding is consistent with the model hypothesis.

### 3.5. Orientation performance in the natural habitat

When allowed to form a ball and roll it away from a dung pat under a clear, sunlit sky in their natural habitat (figure 6*a*), the smaller species, *Scarabaeus ambiguus*, buried their dung balls 7.56 m ± 1.05 m (*N* = 10) away from the pat. This is significantly closer to the pat than the average radial distance travelled by the larger species before burial (*S. lamarcki*: 12.45 m ± 1.28 m, *N* = 10) (Wilcoxon rank-sum test; *p* = 0.010, *Z* = 2.57) (figure 6*b*). Interestingly, the average total distances rolled to reach these burial spots did not differ between the two species (*S. ambiguus*: 20.43 m ± 4.54 m, *N* = 10, *S. lamarcki*: 18.66 m ± 1.94 m, *N* = 10) (Wilcoxon rank-sum test; *p* = 0.85, *Z* = 0.19). This suggests that there is a behavioural mechanism to compensate for the increase in tortuosity that the smaller beetles unavoidably seem to experience (figure 6).

## 4. Discussion

### 4.1. Maintaining a direction in the absence of external visual cues

When deprived of all visually mediated compass cues, the dung beetles failed to maintain a straightforward course and instead moved along tortuous paths (figure 3). Unsurprisingly, spiders, amphibians and humans are also unable to move along a given bearing in the dark [10,38,39]. Under these circumstances, these animals are expected to travel by means of a *correlated random walk* (*CRW*) [33], where each step is intended to point in the same direction as the previous one. This is also the case for the dung beetle, as captured by the *biased correlated random walk model* in the limit *w* = 0 (i.e. with no external compass input, and thus only the second term in equations (2.1) and (2.2) contributes to the accumulated error). Assuming that only internal sensory information was available to these beetles when orienting in the dark, a directional error, most likely caused by mechanosensory noise in the muscles of their moving limbs, accumulated with every step taken. Thus, the instantaneous angular error arising from the accumulated noise in motoric execution is determined as equivalent to the *motor error*. A direct analysis of the direction of each subsequent step when rolling in complete darkness further reveals that this motor error lies at around 30° per step, irrespective of species (*S. ambiguus*: 33.11° ± 5.12°, *S. lamarcki*: 29.41° ± 9.92°). As can be expected, these findings showed that over the same radial distance (50 cm) the smaller *S. ambiguus* (with more steps taken per distance travelled) rolled its ball along a significantly more tortuous trajectory compared to the larger, *S. lamarcki* (figure 3). Tortuous paths can also be observed outdoors, under overcast skies, or when the beetle is prevented from seeing the sky by the use of a cap [12]. Under these conditions, just as in the dark, the beetle cannot access any external visual compass cues to correct for the accumulation of errors in its orientation system.

### 4.2. Dung beetles rely primarily on the sun for straight-line orientation

From the trajectories presented in figure 5 it is evident that, under the open sky, both species of dung beetles orient along straight paths in a given direction, presenting a clear contrast to the more tortuous trajectories taken in the absence of external visual cues (figure 3). The large change in roll bearing recorded for *Scarabaeus ambiguus* and *S. lamarcki* in response to a 180° displacement of the sun (figure 2) demonstrates the common use of a sun compass in these species during straight-line orientation. These results are well in line with previous studies of the celestial compass system of *S. lamarcki* [6,27–30].

When rolling repeatedly to the edge of the 50 cm diameter arena, under an open sky, the larger *S. lamarcki* was significantly better oriented than its smaller relative *S. ambiguus* (figure 5*a*). This difference in performance no longer prevailed when the orientation performance over a radius proportional to 20 steps of the two species (*S. ambiguus*: 32.38 cm, *S. lamarcki*: 51.79 cm) was considered. This again suggests that both species gain a similar sized error with every step taken.

Interestingly, the error that is accumulated while rolling seems not to accumulate over the course of 20 consecutive rolls, as no difference in straightness was found between the first and last roll for either of the species while rolling under an open sky (figure 5). This clearly demonstrates that the error generated by each step taken, while the beetle is rolling its ball, remains the same size irrespective of the number of steps taken.

### 4.3. A biased correlated random walk supports straight-line orientation in dung beetles

To understand the effect of step size on straight-line orientation [34,35], and to model how much weight is given to external sky compass cues over internal proprioceptive cues for straight-line orientation in dung beetles, we chose to connect a vector-weighted *biased correlated random walk model* for directed movement, where external cues are balanced with internal ones [34,35]. The model assumes that the beetle intends to move in a straight line, which is what we have observed in this and many earlier studies of dung beetle orientation (figure 5*b*) [6,12,26–32].

### 4.4. The weighting of different cues provides the best possible compass strategy

The values for the angular error generated in the absence of visual cues, determined as equivalent to the motor error (33° for *S. ambiguus* and 29° for *S. lamarcki*), were used as input parameters to the biased correlated walk model, allowing us to estimate the balance between a *biased random walk* (*BRW*) and a *CRW* employed by a beetle when orienting outdoors. From this model, we can also describe the compass error generated with each step in the two species of dung beetles.

When the beetles are rolling outdoors, under the full view of a natural sky, the weight given to external cues over internal cues is significantly shifted towards external cues (*w_S. ambiguus_* = 0.84 and *w_S. lamarcki_* = 0.83), revealing that the paths of the beetles, irrespective of beetle size, are primarily dictated by a *BRW*. This balance did not differ between the species.

Interestingly, our model further reveals that the compass error is remarkably smaller than the motor error (compass error: *S. ambiguus*: 5.94° and *S. lamarcki*: 6.34° versus motor error: *S. ambiguus*: 33.11° and *S. lamarcki*: 29.41°) with no significant difference in the size of the error between the two species. This difference in angular error, or ‘noise’, generated by the two sources of information (motor and compass) provide a possible explanation for the shift towards external cues by the compass when orienting outdoors.

In parallel to the weighting of external visual cues over internal proprioceptive cues by the dung beetle, ants also seem to rely more heavily on the cue that currently provides the more precise directional information [16]. If directional information from the path integrator (PI) of the ant and the visual scene are set in conflict, the weighting towards the PI will increase as the ants move further from their nest, and their PI vector becomes increasingly longer.

In summary, our results suggest that (i) the analysis systems of the compass cues (visual system and neuronal system) of the smaller beetle are as precise as that of the larger beetles and that (ii) the compass system of the smaller beetles (as in the balance between a *CRW* and a *BRW*) is not specifically evolved to compensate for the directional challenges that arise due to differences in stride length.

### 4.5. The effect of step size on straight-line orientation in the natural habitat

A recent study on ants shows that the ability to orient using compass information is the same across ants that differ in size by a factor of three, but similar to this study, the accumulation of errors increases with the number of steps taken [40]. Consequently, smaller ants, just like the beetles, can be observed to move over more tortuous paths than their larger relatives. Together, these studies nicely demonstrate that the ‘step size error’ has an effect on the ability of these insects to maintain a straight bearing even under an open sky, suggesting that regardless of the availability of an external visual compass cue, this noise cannot be fully compensated for and will work to the disadvantage of the smaller species. The recorded accumulation of error with distance rolled in the beetles is most likely partly due to mechanosensory noise in the motor system when executing each step, and partly due to noise in the acquisition and processing of the celestial compass cues that direct the steps taken [41].

Smaller insects with smaller steps risk travelling along more tortuous, and thus more energetically costly paths, compared to their larger relatives. But, this is only a disadvantage if the orienting insects—regardless of size—aim to travel the same distance. For the beetles, this is not the case. The smaller beetles tended to bury their balls closer to the dung pat, compared to the larger beetles within the same terrain (figure 6*b*). The phenomenon of smaller sized individuals travelling shorter distances than their larger peers, is not uncommon [42–45], on the contrary, there is a strong positive correlation between the distance an animal travels and its body size. In the case of the beetle, it is still unknown how it measures the distance travelled, but the impact of the relative speed at which angular errors accumulate might play a role in this behavioural outcome, resulting in smaller beetle species reaching shorter effective distances with their balls of dung. This will be the focus of future studies.

## 5. Conclusion

Our results show that for an orienting ball-rolling beetle, an angular error accumulates over each step in the absence as well as in the presence of external visual compass cues. Consequently, smaller insects, with proportionally shorter legs, will produce a larger directional error over the same distance travelled. Our results further imply that the nature of the compass systems of different sized insects is not specifically evolved to compensate for the size (step size) of the animal.

## Supplementary Material

Raw data for the behavioural experiments performed

## Data accessibility

The datasets supporting this article are available as electronic supplementary material.
