# Correction: The effect of step size on straight-line orientation

**DOI:** 10.1098/rsif.2024.0580

**Published:** 2024-10-30

**Authors:** Lana Khaldy, Orit Peleg, Claudia Tocco, L. Mahadevan, Marcus Byrne, Marie Dacke

**Affiliations:** ^1^ Lund University, Lund, Sweden; ^2^ University of Colorado Boulder, Boulder, Colorado, USA; ^3^ Santa Fe Institute, Santa Fe, New Mexico, USA; ^4^ University of the Witwatersrand Johannesburg, Johannesburg, South Africa; ^5^ Harvard University, Cambridge, Massachusetts, USA

**Keywords:** orientation, navigation, step size, dung beetle, compass, random walk

*J. R. Soc. Interface* **16**: 20190181. (Published online 07 August 2019). (http://dx.doi.org/10.1098/rsif.2019.0181)

**The error:** We have identified an error in implementing the weighting variable *w* in the biased-correlated random walk (BCRW) model equations (2.1) and (2.2). This error occurred in the computational implementation of the BCRW model. As noted below, the error affected the results and conclusions of the article.

**How the error impacts the results:** The correct implementation should balance the error arising from noise in external visual cue acquisition θ*^BRW^ and the error from accumulated noise in motoric execution θ*^CRW^ with *w*. However, the published ranges for θ*^BRW^ and *w* were inaccurately narrow. The correct bounds are 1.8° ≤ θ*^BRW^ ≤ 74.7° and 0.225 ≤ *w* ≤ 1 for *S. ambiguus*, and 1.8° ≤ θ*^BRW^ ≤ 74.9° and 0.2 ≤ *w* ≤ 1 for *S. lamarcki*, as illustrated in figure C1.

**Figure C1.**
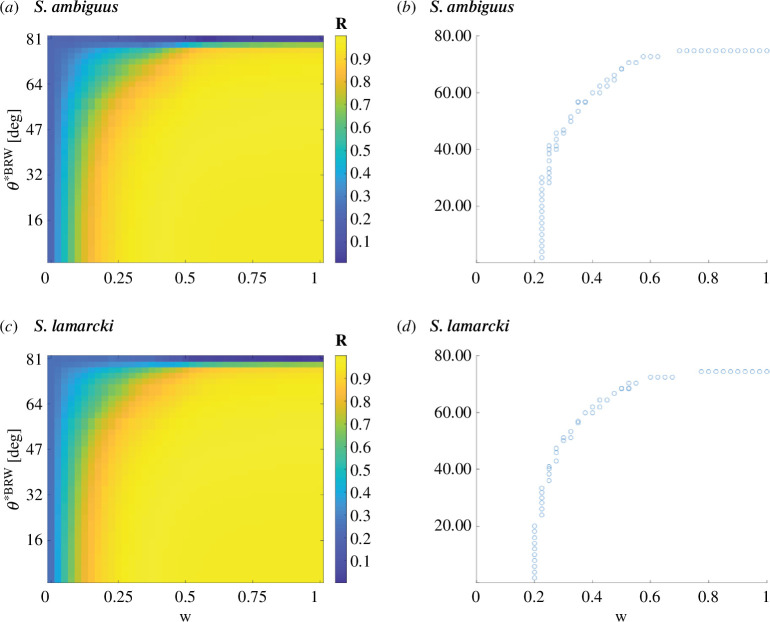
Illustration of revised parameter ranges for θ*^BRW^ and *w* in the BCRW model. (*a,c*) Depict heatmaps showing the relationship between the weighting variable *w* and the error from noise in external visual cue acquisition θ*^BRW^ over the newly established broader range. The colour gradient represents the *R* value, which is the mean vector length. (*b,d*) Provide scatter plots of the identified θ*^BRW^ and *w* pairs, identified by comparing *R* values from simulations and experiments.

**How the error impacts the conclusion:** These expanded ranges suggest that dung beetles exhibit a high degree of flexibility in choosing orientation cues, challenging the original conclusion that they rely predominantly on a sun compass. We believe the experimental data presented remains valuable and that this correction will allow for a more accurate understanding of the orientation mechanisms in dung beetles. Further research is warranted to explore the dynamic nature of the weighting variable *w*.

For completion, we include a revised table 1 and figure 4*b*, Abstract, §§4.3–4.5, and the conclusion of the original paper at the end of this document. The corrected code and simulation results are available at github.com/peleg-lab/rsif.2019.0181_correction. We acknowledge Drs Yakir Gagnon and Chantal Nguyen for identifying and correcting the error.

## 1. Corrections to: Abstract

We have added the following sentence at the end of the abstract:

‘We conclude that the directional error that unavoidably accumulates as the beetle travels is inversely proportional to the step size of the insect, and that both beetle species exhibit a high degree of flexibility in choosing orientation cues.’

## 2. Corrections to: Figure 4

Below are the revised parts of the original figure 4 and table 1.

**Figure 4.**
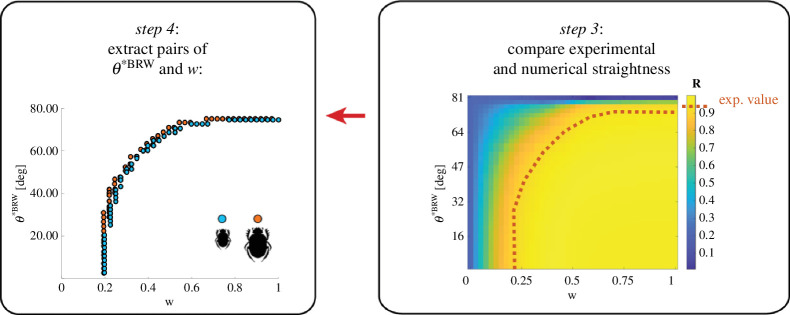
Steps 3 and 4.

**Table 1.** Last rows 3 and 4.

parameter	meaning	experimental value	theoretical value
*w*	balance between CRW and BRW	unknown	extracted to fit properties of experimental trajectories (*R*): *S. ambiguus:* 1.8°≤θ*^BRW^≤74.7°, 0.225 ≤ *w* ≤ 1 *S*. *lamarcki:* 1.8°≤θ*^BRW^≤74.9°, 0.2 ≤ *w* ≤ 1
θ*^BRW^	standard deviation of compass error	unknown	

## 3. Corrections to: Section 3.4

In §3.4, we have amended the text as follows: ‘The angular error generated by each step in the absence of external visual compass cues was introduced as an estimation of motor error (θ*^CRW^) in the biased correlated random walk model [36] (figure 4*b*, step 1). This allowed us to compare the resulting mean vector length, *R*, of the modelled data against the experimentally obtained *R* values (figure 4*b*, step 3) to estimate pairs of *w* (the balance between CRW and BRW) and θ*^BRW^ (standard deviation of compass error) for the two species (figure 4*b*, step 4). From this, we extracted the bounds as 1.8° ≤ θ*^BRW^ ≤ 74.7° and 0.225 ≤ *w* ≤ 1 for *S. ambiguus*, and 1.8° ≤ θ*^BRW^ ≤ 74.9° and 0.2 ≤ *w* ≤1 for *S. lamarcki*. This suggests that both beetle species exhibit a high degree of flexibility in choosing orientation cues.’

## 4. Corrections to: Section 4.4

We have replaced the text in §4.4 as follows: ‘The values for the angular error generated in the absence of visual cues, determined as equivalent to the motor error (33° for *S. ambiguus* and 29° for *S. lamarcki*), were used as input parameters to the biased correlated walk model, allowing us to estimate the balance between a biased random walk (BRW) and a CRW used by a beetle when orienting outdoors. From this model, we can also describe the compass error generated with each step in the two species of dung beetles, and how it is balanced with the motor error. We found that both beetle species exhibit a high degree of flexibility in choosing orientation cues, bounded by 1.8° ≤ θ*^BRW^ ≤ 74.7° and 0.225 ≤ *w* ≤ 1 for *S. ambiguus*, and 1.8° ≤ θ*^BRW^ ≤ 74.9° and 0.2 ≤ *w* ≤ 1 for *S. lamarcki*.’

## 5. Corrections to: Conclusion

We have added the following text to the conclusion: ‘Our results further imply that both beetle species exhibit a high degree of flexibility in choosing orientation cues. Further research is warranted to explore the dynamic nature of the weighting variable *w*.’

